# Deciphering a hexameric protein complex with Angstrom optical resolution

**DOI:** 10.7554/eLife.76308

**Published:** 2022-05-26

**Authors:** Hisham Mazal, Franz-Ferdinand Wieser, Vahid Sandoghdar

**Affiliations:** 1 Max Planck Institute for the Science of Light Erlangen Germany; 2 Max-Planck-Zentrum für Physik und Medizin Erlangen Germany; 3 Friedrich-Alexander University of Erlangen-Nürnberg Erlangen Germany; Institute of Photonic Sciences Spain; Goethe University Germany

**Keywords:** cryogenic super-resolution, correlative imaging, protein structure, assembly, fluorescence, None

## Abstract

Cryogenic optical localization in three dimensions (COLD) was recently shown to resolve up to four binding sites on a single protein. However, because COLD relies on intensity fluctuations that result from the blinking behavior of fluorophores, it is limited to cases where individual emitters show different brightness. This significantly lowers the measurement yield. To extend the number of resolved sites as well as the measurement yield, we employ partial labeling and combine it with polarization encoding in order to identify single fluorophores during their stochastic blinking. We then use a particle classification scheme to identify and resolve heterogenous subsets and combine them to reconstruct the three-dimensional arrangement of large molecular complexes. We showcase this method (polarCOLD) by resolving the trimer arrangement of proliferating cell nuclear antigen (PCNA) and six different sites of the hexamer protein Caseinolytic Peptidase B (ClpB) of *Thermus thermophilus* in its quaternary structure, both with Angstrom resolution. The combination of polarCOLD and single-particle cryogenic electron microscopy (cryoEM) promises to provide crucial insight into intrinsic heterogeneities of biomolecular structures. Furthermore, our approach is fully compatible with fluorescent protein labeling and can, thus, be used in a wide range of studies in cell and membrane biology.

## Introduction

Proteins and their various assemblies are among the main constituents of all living systems and govern every aspect of cellular physiology in both healthy and disease states (Nelson, 2017; Mavroidis et al., 2004; Schliwa and Woehlke, 2003; Alberts, 1998). These biomolecular structures adopt sophisticated three-dimensional (3D) configurations during their multifaceted conformational changes. A full understanding of their spatial arrangements and associated heterogeneous configurations is crucial for elucidating their molecular mechanisms, helps guide the engineering of new proteins, and is a great asset for drug discovery (Renaud et al., 2018). Indeed, since the pioneering work of Perutz on protein crystals (Fersht, 2008), a variety of techniques such as X-ray crystallography (Shi, 2014) and nuclear magnetic resonance (NMR) spectroscopy (Kanelis et al., 2001) have been explored for gaining insight into protein structure and function. Advances in sample preparation, detector technology, and image processing based on single-particle analysis have also ushered in atomic resolution in cryogenic electron microscopy (cryoEM) studies of protein structure (Nakane et al., 2020; Kühlbrandt, 2014). The inherent resolution of this method is highly desirable, but lack of specific labeling makes it challenging to identify small variations such as structural inhomogeneities. Fluorescence microscopy, on the other hand, draws its success from an exquisite specificity in labeling, but has traditionally suffered from a limited resolution.

The recent advent of super-resolution (SR) fluorescence microscopy has opened new avenues for studying subcellular organization and is on the way to become a workhorse for biological studies (Lelek et al., 2021; Sahl et al., 2017; Weisenburger and Sandoghdar, 2015). However, conventional SR microscopy performed at room temperature is still not considered as a contestant in the arena of structural biology, where Angstrom-level information about the molecular architecture of proteins and protein complexes is sought after. To push the limit of fluorescence microscopy, one can perform measurements under cryogenic conditions (Böning et al., 2021, Hoffman et al., 2020; Dahlberg et al., 2020; Moser et al., 2019; Wang et al., 2019; Hulleman et al., 2018b, Xu et al., 2018; Weisenburger et al., 2017; Furubayashi et al., 2017; Li et al., 2015; Weisenburger et al., 2014; Weisenburger et al., 2013). In addition to slowing down photochemistry, which allows each fluorophore to emit several orders of magnitude more photons than at room temperature (Li et al., 2015; Weisenburger et al., 2014; Weisenburger et al., 2013), a key advantage of cryogenic temperatures is in offering superior sample preservation and high stability for Angstrom-scale structural studies. In one implementation, cryogenic optical localization in 3D (COLD) was introduced, where the stochastic intensity blinking of organic dyes gave access to the positions of up to four labeling sites on a single protein (Weisenburger et al., 2017). We recently employed a more robust protocol to identify individual fluorophores by exploiting the polarization of the fluorescence light dictated by the fluorophore orientation (Böning et al., 2021). This latter method was validated by measuring single distances on one-dimensional DNA nanorulers (Böning et al., 2021). Control of the fluorescence signal via polarization modulation has also been shown to offer an alternative to random blinking (Hulleman et al., 2018a, Hafi et al., 2014). In our current study, we introduce polarCOLD, which exploits polarization encoding for resolving several fluorophores in 3D. Importantly, we show that the distances and arrangements of protein complexes can be determined by combining images recorded from under-sampled structures. To demonstrate this, we first resolve three fluorophores on a trimer protein complex with Angstrom resolution. Next, we use partial labeling, a supervised particle classification procedure to solve a complete hexameric protein arrangement. We discuss the limits of our methodology for resolving structures with a certain degree of disorder as well as its promise for combination with cryoEM.

## Results

### polarCOLD on a protein trimer

Proliferating cell nuclear antigen (PCNA) is a central functional unit in genome repair and replication (Bruck and O’Donnell, 2001). The structure of this complex protein was solved by X-ray crystallography (Georgescu et al., 2008) and more recently by cryoEM (Madru et al., 2020). These studies have shown that PCNA forms a stable homo-trimer with a pseudo-hexameric shape. The stability and simple configuration of its structure make PCNA a good model system for benchmarking our imaging methodology. To study human PCNA with polarCOLD, we first fully labeled it via a His-tag linker on the N-terminal side of each subunit of the protein, forming an equilateral triangle (see Materials and methods section and Figure 1—figure supplement 1). PCNA complexes were then embedded in a hydrophilic poly-vinyl alcohol (PVA) matrix at sub-nanomolar concentration and spin-coated on a mirror-enhanced substrate (see Böning et al., 2021 for in-depth characterization of the mirror-enhanced substrate). The resulting density corresponds to fewer than one protein per μm^2^ on average so that individual proteins can be easily identified in diffraction-limited imaging. The samples were immediately imaged in our custom-built microscope (see Materials and methods section).

**Figure 1.**
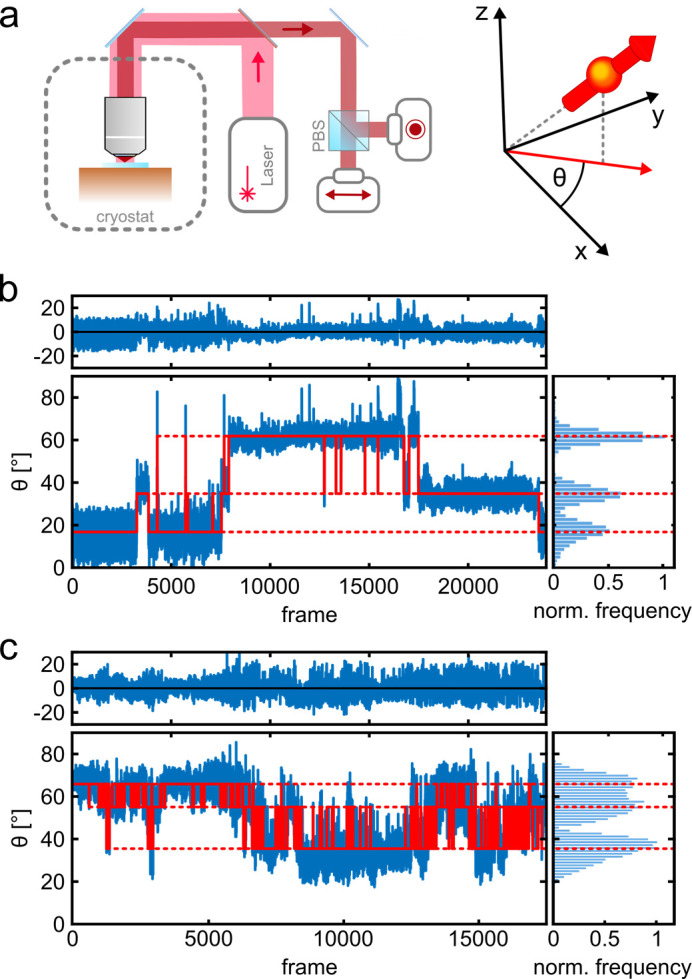
Photo-physics and co-localization of fluorophores at cryogenic temperatures. (**a**) Schematics of the cryogenic optical microscope. Polarization-resolved detection allows for direct measurement of the in-plane dipole moment of fluorophores. Here we use circularly polarized light from a laser at λ=635 nm. A polarizing beam splitter in the detection path allows one to resolve the polarization state of each individual molecule. (**b, c**) Exemplary polarization time traces of two single proteins. (**b**) demonstrates a case of well-separated polarization states, whereas (**c**) displays a case with smaller separations between polarization states. Blue traces present the experimental polarization values for each frame, and the red lines show the polarization determined by the algorithm (White et al., 2020). Top panels shows the residuals of the fit. The blinking kinetics are exceptionally slow with on/off times in the range of seconds to minutes. Figure 1—source data 1.Exemplary polarization time traces of two single proteins for Figure 1b-c. Figure 1—source data 2.Overview of the recorded data and the experimental yield.

Figure 1a shows a schematic of the imaging setup. A liquid helium cryostat houses the cold stage as well as a scannable microscope objective (Weisenburger et al., 2017; Böning et al., 2021). A polarizing beam splitter in the detection path allows us to determine the orientation of dipole-like emitters projected onto the angular interval θ ϵ [0°, 90°] in the imaging plane. The high photostability of the fluorophores at T=4 K allows us to collect on average 260 photons per frame (per 14 ms) from a single fluorophore with a total number of registered photons exceeding 10^6^ after 50,000 frames (Figure 1—figure supplement 2). Figure 1b–c (blue trace) displays two examples in which three different polarization states recur at various times. The long photo-blinking off-times (Figure 1—figure supplement 2d) allow one to identify each of the three fluorophores on a given protein complex separately (Weisenburger et al., 2017). More insight into polarization trace processing can be found in Figure 1—figure supplement 3. To resolve the polarization histograms in cases where they partially overlap (see, e.g., Figure 1c), we fit the data using an algorithm that combines unsupervised statistical learning tools with change-point detection in a model-independent manner (White et al., 2020). As illustrated by the red traces in Figure 1b and c, we can robustly identify the polarization states over time and hence assign the signal in each frame to a single fluorophore. We also verified the robustness of our assignment procedure by performing random assignment of frames, which resulted in single, unresolved spots (see Materials and methods section and Figure 1—figure supplement 4).

**Figure 1—figure supplement 1.**
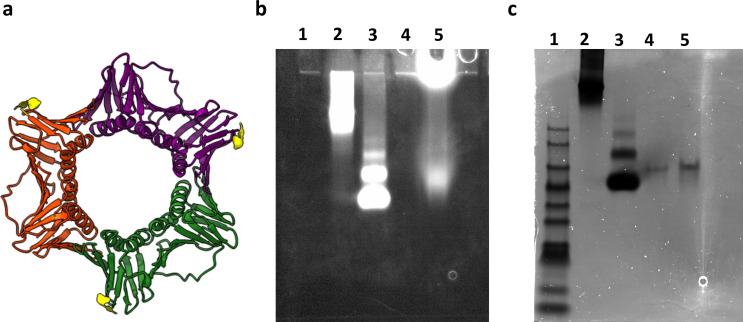
Characterization and labeling of human PCNA protein. (**a**) Schematics of the human PCNA crystal structure (PDB: 1AXC). Each monomer is presented in a different color (green, red and purple). The location of the N-terminal His-tag is shown in yellow. Native gel electrophoresis (4–15% Mini-PROTEAN TGX Precast Protein Gels) ran in 25 mM Tris and 192 mM glycine, pH 8, at 60 V for 3 hr. The gel was imaged with a ChemiDoc XRS system at 647 nm (**b**) and was then stained with Coomassie Blue (**c**). Lane 1 is the protein ladder PM2500 (SMOBIO), lane 2 is thyroglobulin from bovine serum labeled with ATTO647N-NHS, lane 3 is Bovine serum albumin protein (BSA) labeled with ATTO647N-NHS, lane 4 is the non-labeled human PCNA, lane 5 is human PCNA labeled with ATTO647N-NTA. Lanes 4 and 5 of human PCNA in the gel show only one band indicating a homogenous and pure solution of the assembled proteins. Also, this result shows that the labeled human PCNA ran similar to the non-labeled one, indicating that the dye did not affect the protein. Figure 1—figure supplement 1—source data 1.Raw Native-page and fluorescent gel images.

**Figure 1—figure supplement 2.**
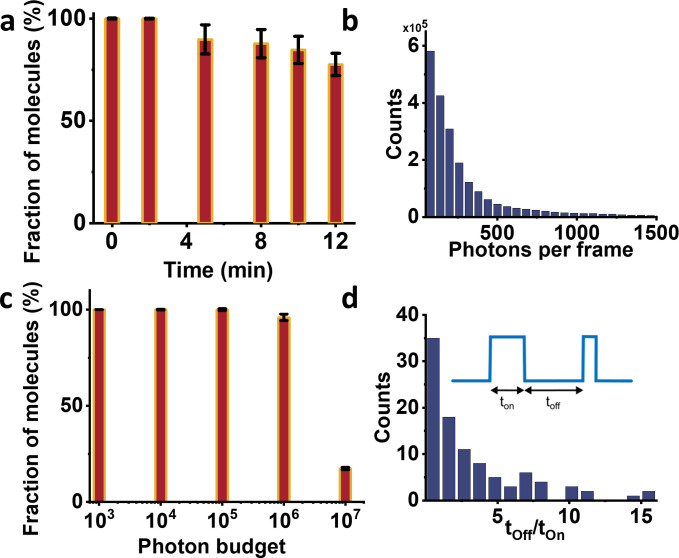
Photo-physics characterization of NTA-ATTO647N attached to human PCNA protein imaged at 4K. (**a**) Fluorophore stability histogram shows the fraction of molecules which survived until a given time of data acquisition. Most molecules survived until the end of the measurement, allowing improved photon collection and higher localization precision. (**b**) Histogram of the mean number of photons per frame. On average we obtain ~260 photons per frame (per 14 ms). (**c**) Photon budget histogram plotted as a fraction of molecules versus total number of photons collected from 50,000 frames. Around 17% of the molecules emitted at least 10^7^ photons. However, this number is mostly limited by the shorter recording time in these measurements and can be increased further if necessary. (**d**) The photo-switching behavior of the fluorophores is characterized by the dwell times in the off- and on-state. The ratio of their average duration indicates long-lived off-state facilitate localizing single emitters via polarization-trace fitting. Error bars in a and c represent standard errors of the mean.

**Figure 1—figure supplement 3.**
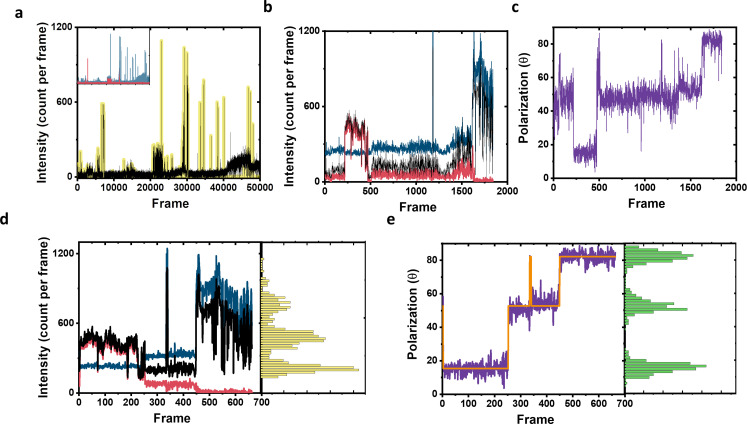
Example of complete intensity and polarization trace processing for fluorophore localization. (**a**) Raw total photon counts registered from the two channels (black line). Yellow highlights show the regions which were determined as on events and taken for further analysis. The inset shows the photon signal in each channel separately shown in blue and red. (**b**) Intensity time trace of the on events highlighted in (**a**). The blue and red lines represent the intensity of the two polarization channels, and the black line is the total signal. For clarity, the blue line was shifted 200 counts on the y-axis. (**c**) Polarization trace (purple color) of the signal calculated from (**b**). (**d**) The signal trace after filtering based on photon counts per frame (frames below 100 photons were excluded) with the yellow histogram on the side showing the photon distribution over the whole measurement. The color code is the same as in (**b**). For clarity, the blue line was shifted 200 counts on the y-axis. (**e**) The polarization trace (purple color) as calculated from intensity signal shown in (**d**) and used for fluorophore assignment and further analysis. The green histogram on the side shows the polarization distribution. The Orange solid line is the fit of the polarization trace as calculated using DISC algorithm, which combines unsupervised statistical learning tools with change-point detection in a model-independent manner (White et al., 2020). The fit clearly show three separate polarization states, which indicate the fluorophores attached to this molecule. Next, we take the assignment of the polarization trace to assign and localize the coordinate of each fluorophore separately. Figure 1—figure supplement 3—source data 1.Raw data set used as an example for complete signal processing calculated from the same single molecule.

**Figure 1—figure supplement 4.**
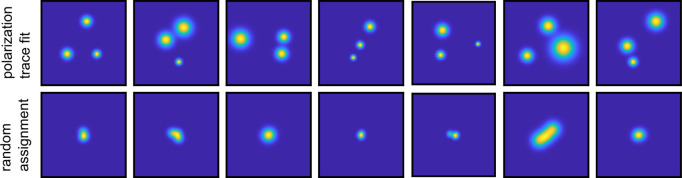
Validation of polarization assignment. (**a**) Example of 2D maps of the fluorophore localization as obtained for each fluorophore based on fitting the polarization trace, as described in Figure 1—figure supplement 3. (**b**) In order to validate the assignment of the detected photons originating from a diffraction-limited spot to a specific fluorophore, we performed random assignment of frames. After averaging these randomly assigned localizations, the super-resolved image shows single spots in the center. In comparison, the polarization time trace fit accurately resolves the three positions in (**a**).

**Figure 1—figure supplement 5.**
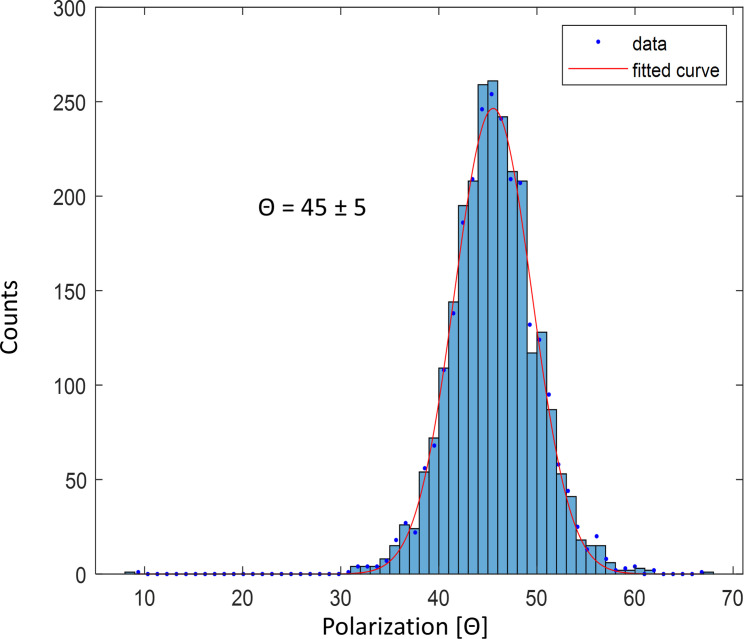
Polarization histogram of single fluorophore ATTO647N imaged at 4 K. The histogram shows a single peak with a width of 5 degrees, which matches the expected shot noise value estimated from 100 photons.

**Figure 1—figure supplement 6.**
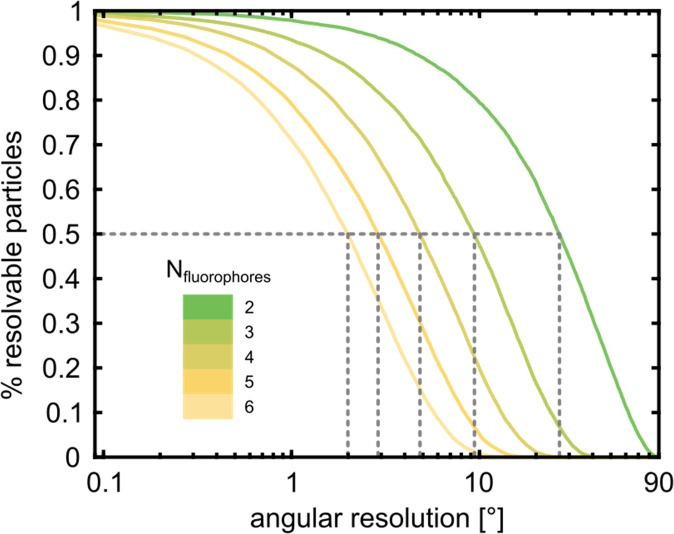
Fluorophore identification using polarization-resolved imaging. The fraction of particles for which all fluorophores can be identified by the analysis of their polarization time-trace is shown as a function of the angular resolution of the fit. In order to resolve 50% of a hexamer sample with 6 fluorophores, an angular resolution of 2° is required. However, to resolve a partially labeled hexamer with 3 fluorophores only requires a resolution of 10°. For this simulation, we assumed random dipole angles and accounted for the mapping into the [0°, 90°] interval (see Materials and methods section).

The number of fluorophores that can be simultaneously resolved depends on the blinking on-off dynamics (see Materials and methods section). Furthermore, the shot noise determines the angular resolution and, thus, the maximum number of resolvable polarization states per protein. This, in turn, directly affects the yield of resolved particles (see Figure 1—figure supplements 5–6). For example, in our current experiment, we used one polarization basis and projected all orientations to the limited space of θ ϵ [0°, 90°]. By taking the experimental angular resolution of ca. 5° (Figure 1—figure supplements 5–6), we can theoretically expect to resolve 70% of the particles which contain 3 fluorophores, and roughly 15% of the particles which contain 6 fluorophores. Adding a second polarization basis at a tilt of 45°, would allow one to double the angular space (Stallinga and Rieger, 2012). Moreover, using polarized illumination (Backer et al., 2016; Zhanghao et al., 2019) and a full 3D characterization of the dipole moments (Lieb et al., 2004; Mortensen et al., 2010; Hulleman et al., 2021) would result in less overlap and thus enhanced capacity for identifying different polarization states. We remark, however, that even in our current scheme, one can improve the number of resolved polarization states by excluding the ambivalent cases, albeit at the expense of the overall yield (see Figure 1—figure supplement 6 and Figure 1—source data 2 for the statistics of this analysis).

**Figure 2.**
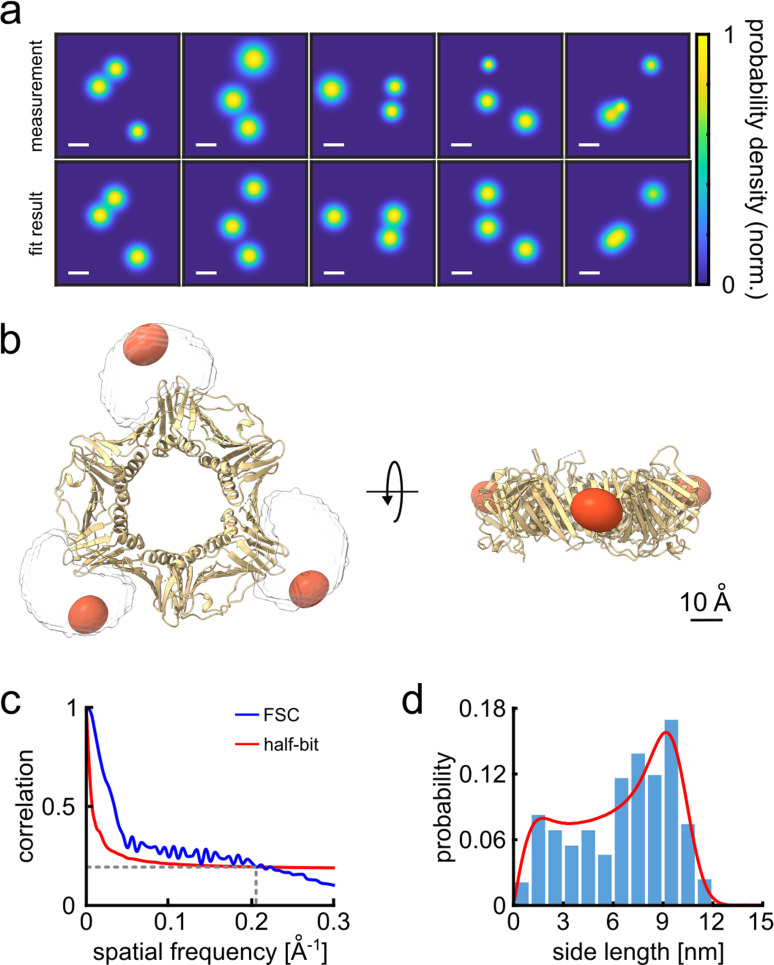
3D reconstruction of the PCNA protein trimer. (**a**) Experimentally obtained super-resolved 2D images (top row) of single proteins and simulated images based on the crystal structure (bottom row). The color code represents the occupation probability determined by the localization precision for each fluorophore. The localization precision in the simulated data was normalized. Scale bar is 3 nm. (**b**) Overlay of the crystal structure of human PCNA with the reconstructed fluorophore volumes shown as red spheroids (see online Animation 1). The transparent white clouds represent the accessible volume of the dye linker attached to the N-terminal side of the protein, calculated using the parameter of ATTO647N as provided in Kalinin et al., 2012. By fitting the reconstructed 3D volumes obtained from polarCOLD into the theoretical accessible volumes of the dyes, we find a correlation score of 0.96, indicating a correct 3D reconstruction. (**c**) The Fourier shell correlation (FSC, blue curve) of the two half data sets gives a resolution of 4.9 Å based on the half-bit criterion (red curve). (**d**) Distribution of the projected side lengths (blue) obtained from the localized positions shown in (**b**). The model fit (red) takes the finite localization uncertainty and the random particle orientation into account, resulting in 9.9±0.6 nm. The error of the model fit was estimated from 200 fits. The reconstructed 3D volume was calculated from 119 particles. Figure 2—source data 1.Full dataset coordinates of the 2D images used for 3D reconstruction for Figure 2a. Figure 2—source data 2.Human PCNA 3D reconstructed map for Figure 2b. Figure 2—source data 3.Fourier shell correlation data for Figure 2c. Figure 2—source data 4.Distance histogram data and model fit for Figure 2d.

Having identified the individual fluorophores, we generate super-resolved images by clustering the respective coordinates and taking their averages (see Figure 2—figure supplements 1 and 2 for moving from traces to 2D resolved image). The top and bottom rows in Figure 2a display a selection of the measured and simulated 2D projection maps. To quantify their similarity, we computed a correlation score ranging from 0 to 1. We obtained 2D correlation scores of 0.92 or higher, representing nearly perfect agreement. To obtain a 3D model from our 2D localization maps, we use a single-particle reconstruction algorithm (Dvornek et al., 2015; Weisenburger et al., 2017; see Materials and methods section and Figure 2—figure supplement 3 for complete data set). Figure 2b shows that the reconstructed fluorophore volumes (red spheroids) agree well with the crystal structure of the PCNA protein (PDB: 1AXC) containing three identical subunits in an equilateral triangle. The slight asymmetry and deviation from the actual crystal structure can in part be attributed to the uncertainty introduced by the dye linker, 6-histidine linker and possibly the restricted rotational mobility of the dye itself, resulting in a minor localization bias. Indeed, by taking the dye linker into account and calculating the accessible volume (Kalinin et al., 2012), we found that our 3D reconstructed volumes correlate very well (0.96 correlation score) with the simulated accessible volumes (see Figure 2b, Animation 1).

**Figure 2—figure supplement 1.**
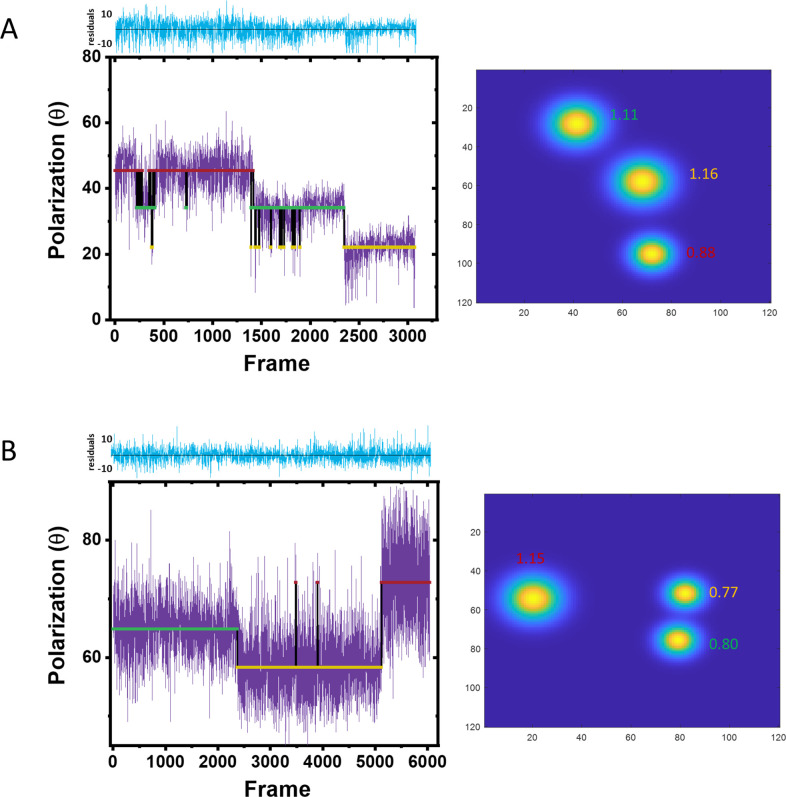
From polarization trajectories to 2D images. (**A–B**) Two different trajectories with three assigned polarizations. Each polarization state was colored differently and shown as red, green, and yellow line, respectively. The localization precision (nm) of each fluorophore is indicated on the 2D super-resolved image accordingly.

**Figure 2—figure supplement 2.**
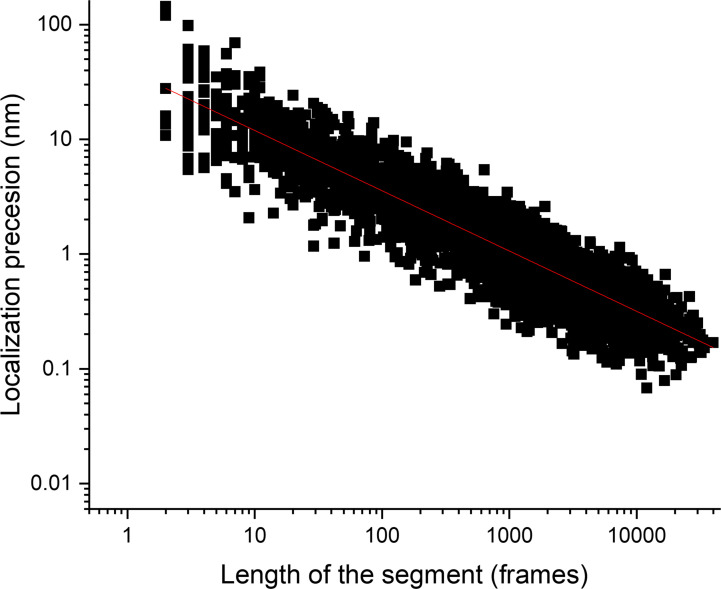
Length of the segment and localization precision. Plot of the localization precision as a function of the segment length, plotted as a log-log plot. The figure shows the increase in localization precision with the length of the segment.

**Figure 2—figure supplement 3.**
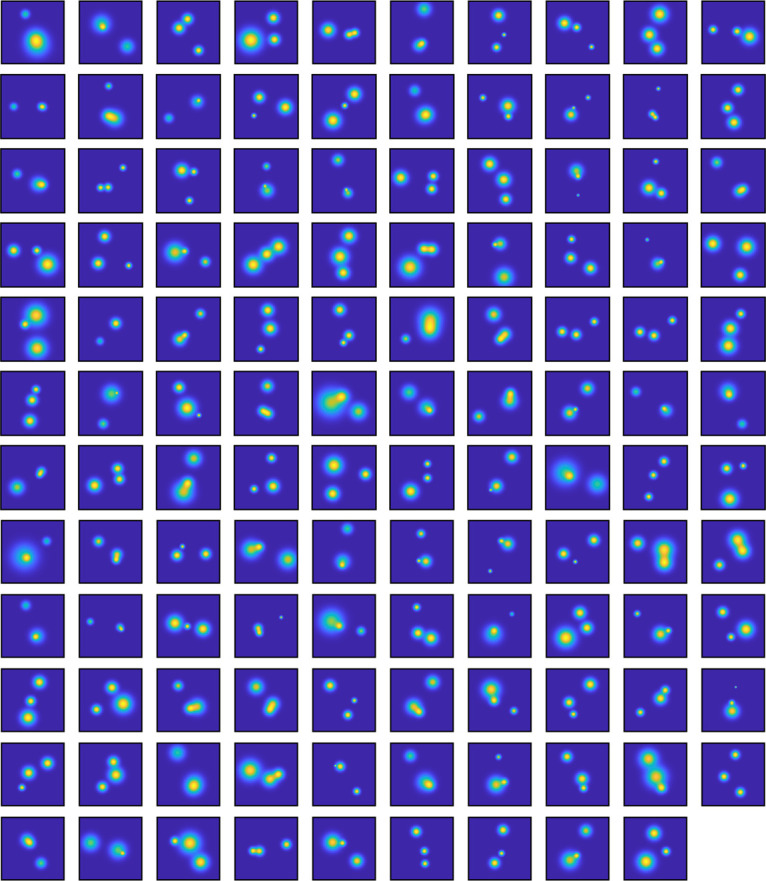
Complete data set of the PCNA protein trimer used for 3D reconstruction. All 2D maps as obtained from polarization trace fitting with a three-state model. Particles were filtered based on localization precision below 3 nm. The image size is 120 × 120 pixel at 0.15 nm/pixel. Figure 2—figure supplement 3—source data 1.Full dataset of normalized center of mass coordinates of the 2D images used for 3D reconstruction.

**Animation 1.** Human PCNA 3D reconstruction.

To evaluate the resolution of our 3D reconstructed volumes, we used the well-established method of Fourier shell correlation (FSC) (van Heel and Schatz, 2005). Here, we divide the 2D image data set into two randomly chosen groups and then determine their 3D reconstruction separately. Then we assess the cross-correlation (similarity) between the two 3D volumes in Fourier space as a function of spatial frequency. The overall resolution of a 3D reconstructed volume is thus obtained by finding the maximum spatial frequency corresponding to a correlation above a specific threshold value. Here, we used the half-bit criterion, which is a standard threshold curve used in single-particle cryoEM. As shown in Figure 2c, the intersection of this curve (red) with the FSC curve (blue) indicates at which spatial frequency we have collected a sufficient amount of information in order to interpret the 3D reconstructed volumes accurately (van Heel and Schatz, 2005). We find a remarkable resolution of 4.9 Å. We further quantified the size of the protein-dye conjugate via the pair-wise distances between the localized sites on each particle. The histogram in Figure 2d plots the distribution of the side lengths of the projected triangles and is well-described by a fit that considers the localization uncertainty as well as the random particle orientation (Böning et al., 2021, Weisenburger et al., 2017). We determine a side length of 9.9±0.6 nm in excellent agreement with the expected value. The uncertainty was determined via bootstrapping and is consistent with the resolution obtained from the FSC curve. We note that the high signal-to-noise ratio (SNR) of the method (see, e.g, Figure 2a), and the comparatively low information density per particle, deliver a good results from a total of 119 particles (see Figure 1—source data 2 for overall statistics), which is two to three orders of magnitude lower than the number required for typical cryoEM measurements (Cheng et al., 2015). Indeed, the low SNR in cryo-EM requires data from a large number of particles to be first averaged to establish 2D classes before using them for 3D reconstruction (Rosenthal and Henderson, 2003). In our case, each 2D projection directly contributes to the 3D reconstruction process.

### Resolving a hexameric protein complex using partial labeling

The trimer structure discussed above involves only a single dye-dye distance. We now turn to resolving an example of more complex higher-order protein structures. Considering that a limited number of fluorophores can be resolved via stochastic blinking (Figure 1—figure supplement 6), we pursued a strategy of partial labeling of the sites of interest on a given individual protein complex. The piece-wise information, which involves various fluorophore arrangements and distances is then assembled to solve for the full architecture using prior knowledge of the symmetry. This concept has been successfully used to build structural models in NMR (Fiaux et al., 2002) and more recently in SR microscopy (Heydarian et al., 2018; Molle et al., 2018). To demonstrate this technique, we examine the homo-hexamer Caseinolytic Peptidase B (ClpB) of *Thermus thermophilus* in its quaternary structure (Lee et al., 2003; Diemand and Lupas, 2006; Figure 3—figure supplement 1a). ClpB is a molecular machine that rescues proteins from aggregation within cells (Doyle et al., 2013), and its structure was shown to be very stable in the presence of ATP at low concentrations (Mazal et al., 2019). Here, we labeled the M-domain of the protein at residue S428C such that the distance between two adjacent labeling sites is expected to be 9 nm after accounting for the dye linker (Figure 3—figure supplement 1a). We deliberately reduced the labeling efficiency to 50% in order to allow for 1/3 of the particles to carry three fluorophores as estimated by the binomial distribution (Figure 3—figure supplement 1b).

Labeled ClpB complexes were imaged in the same fashion as before in the presence of 2 mM ATP to stabilize the protein assembly. We obtained about 510 photons per frame on average and a significantly higher total number of photons after 100,000 frames since 87% of the molecules survived until the end of the acquisition interval (see Figure 3—figure supplement 1c-g for photo-physics charectrization). The photo-blinking behavior was similar to that observed in PCNA with a slight increase in the average off-on ratio, allowing robust fluorophore assignment. Hence, we fitted our polarization traces as described previously and selected only those cases that contained three fluorophores. Particles carrying three labels inherently fall into three distinct classes (I, II, and III) with multiple pair-wise distances of approximately 9, 15, and 18 nm (Figure 3—figure supplement 1a).

**Figure 3.**
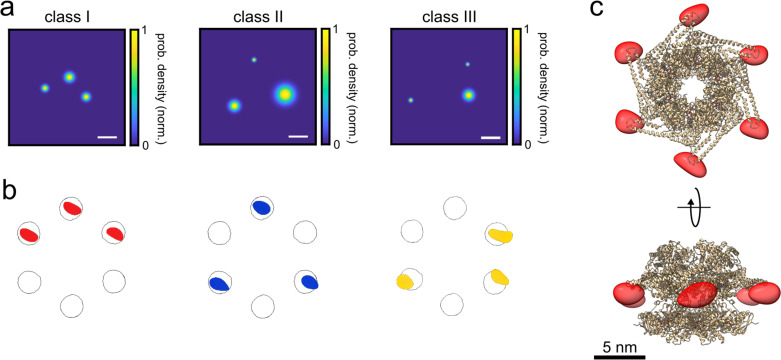
3D reconstruction of the ClpB hexamer protein. (**a**) Top view of super-resolved 2D images for classes I, II, and III, as obtained from single-particle classification procedure. Scale bar is 3 nm. (**b**) Result of single-particle classification and averaging. The reconstructed 3D volume of each class nicely sits in the simulated accessible volume of the fluorophores (grey spheres). Red, blue and yellow spheres represent classes I, II, and III with correlation values of 0.98, 0.96, and 0.86, respectively. (**c**) 3D reconstruction of the complete hexamer obtained from merging the 3D volumes (red spheroid) of the three classes. Top figure shows the top view of the reconstructed 3D volume of the hexamer shape, and the bottom figure shows its 90° rotation (see online Animation 2 and 3Animation 2 and 3). Crystal structure of ClpB is shown as a cartoon in gold (PDB: 1QVR) (Lee et al., 2003; Diemand and Lupas, 2006). Reconstructed 3D volumes were calculated from 232, 100, and 135 particles for classes 1, 2, and 3, respectively. Figure 3—source data 1.Full dataset coordinates of the 2D images used for 3D reconstruction of each class for Figure 3a. Figure 3—source data 2.Full raw dataset coordinates of the unclassified particles for Figure 3a. Figure 3—source data 3.3D reconstituted maps of each class for Figure 3b. Figure 3—source data 4.3D reconstituted maps of the hexamer complex for Figure 3c.

To analyze the resulting triangle images, we used a supervised classification scheme (Gao et al., 2004) to assign each 2D image to one of the three classes, taking the angle of its plane into account. Here, we simulated large data sets of 2D projections for each class and applied a template matching procedure based on the 2D cross-correlation between an experimental image and the simulated images. A control based on simulated ground truth images obtained from the crystal structure of ClpB showed an accuracy of 98% in template matching. Figure 3a displays examples of the 2D super-resolved images of each class (see Figure 3—figure supplement 2 for more examples of different projections, and Figure 3—source data 1 and Figure 3—source data 2 for full dataset). We assigned each image to the class that yielded the highest correlation score. Images that matched all classes with a difference below 10% for class 1 and 3, and below 3% for class 2 in the score (estimated from a simulation analysis, see Figure 3—figure supplement 3) were excluded from further analysis in order to avoid smearing (see Figure 1—source data 2 for all statistics). Next, we picked 2D images of each class with a localization precision better than 3 nm (Figure 3—figure supplement 1c) and correlation score better than 0.9 and calculated their respective 3D structures as described previously for PCNA. Overall, we obtained 232, 100, and 135 particles for classes 1, 2, and 3, respectively, representing a yield of ~7% for all the particles detected with three polarization states (see Figure 1—source data 2). Remarkably, as illustrated in Figure 3b, the reconstructed 3D volumes fit very well to the probable locations of the dye on the protein structure given by the accessible volumes (circles) on each protomer after taking the dye linker into account. The correlation scores between the reconstructed 3D volumes and the accessible volumes of the dyes were 0.98, 0.96, and 0.86 for classes I, II, and III, respectively. As a control, we also performed a reconstruction of unclassified 2D images, and confirmed that no structure was identified (Figure 3—figure supplement 4). Again, the FSC curves of the volumes (see Figure 3—figure supplement 5a-c) suggest an exquisite resolution of 4.0, 7.9, and 6.4 Å, for classes I, II, and III, respectively. Following the successful assignment of the three classes, we merged them as shown in Figure 3c to obtain the complete 3D shape of the hexamer structure (see Animation 2 and 3).

**Figure 3—figure supplement 1.**
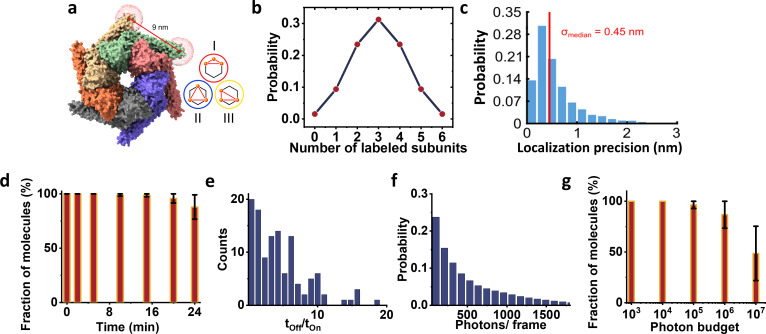
ClpB labeling and photo-physics. (**a**) We modeled the dye positions using the method described previously in Figure 2b. In particular, we calculated the accessible volume of the dye-linker attached to the sulfur group of a cysteine amino acid at position 428. We used dye parameters for ATTO647N maleimide as provided in Kalinin et al., 2012. We used the center of mass of the obtained cloud coordinates to calculate the expected pair-wise distances between fluorophores and obtained a distance of 9 nm between two adjacent subunits. Given the planar hexameric geometry, the other possible pair-wise distances are 15.5 nm and 18 nm. Inset shows the three possible configurations, classes I, II, and III obtained from particles labeled with three fluorophores. (**b**) The fraction of labeled protomers in ClpB molecules was calculated based on the binomial distribution. Here, we assume 50% labeling efficiency and therefore expect that 1/3 of proteins will carry three fluorophores. (**c**) Histogram of localization precisions with a median of 0.45 nm obtained from the hexamer data. (**d-g**) Photo-physics characterization of ATTO647N-maleimide attached ClpB protein imaged at 4 K. (**d**) The fluorophore stability histogram demonstrates a high survival fraction of molecules. (**e**) Histogram of the off-on ratio. (**f**) The mean number of photons per frame shows a twofold increase compared to the PCNA trimer with an average of ~510 photons. (**g**) Photon budget histogram plotted as a fraction of molecules versus total number of photons collected from 100,000 frames. Around 50% of the molecules emitted at least 10^7^ photons. Error bars in d and g represent standard errors of the mean.

**Figure 3—figure supplement 2.**
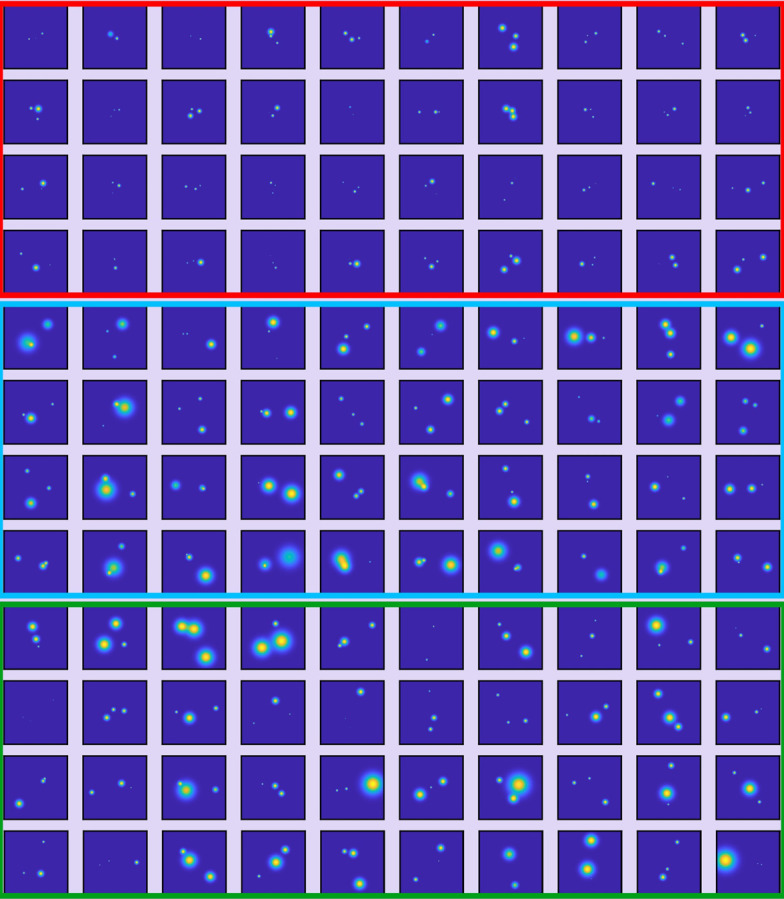
Data set of the ClpB hexamer protein used for classification. Some examples of the resolved 2D maps as obtained from polarization trace fitting with a three-state model. Particles were filtered based on a localization precision below 3 nm and classified (red = class I, blue = class II, green = class III). The image size is 200 × 200 pixel at 0.15 nm/pixel. Figure 3—figure supplement 2—source data 1.Full dataset of normalized center of mass coordinates of the 2D images used for 3D reconstruction.

**Figure 3—figure supplement 3.**
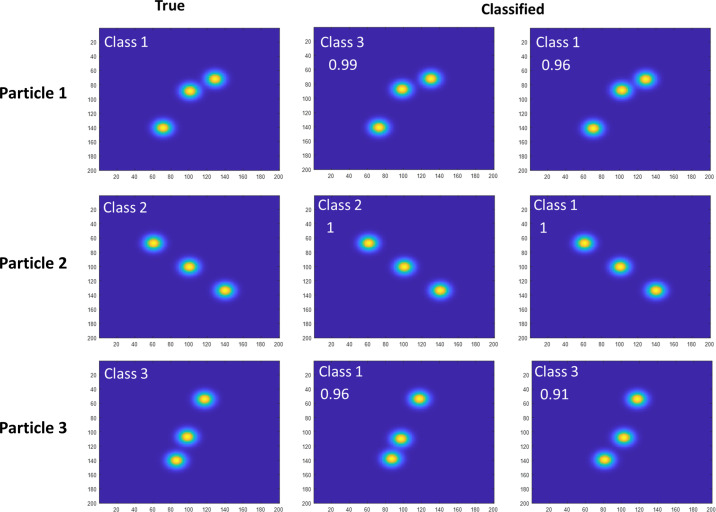
Estimation of particle misclassification based on simulation. Examples of simulated projections from all classes were subject to template matching to evaluate the classification procedure. Results showed that we have up to 2% misclassification. The figure shows representative examples of these 2% of misclassified particles. The top row shows a particle which was put into class 3 rather than class 1. The second row shows a particle which was classified into 1 rather than 2. The third row shows a particle which was classified as 1 rather than 3. The difference between the scores of these particles is below 5%. We exclude such particles in our pipeline to avoid smearing.

**Figure 3—figure supplement 4.**
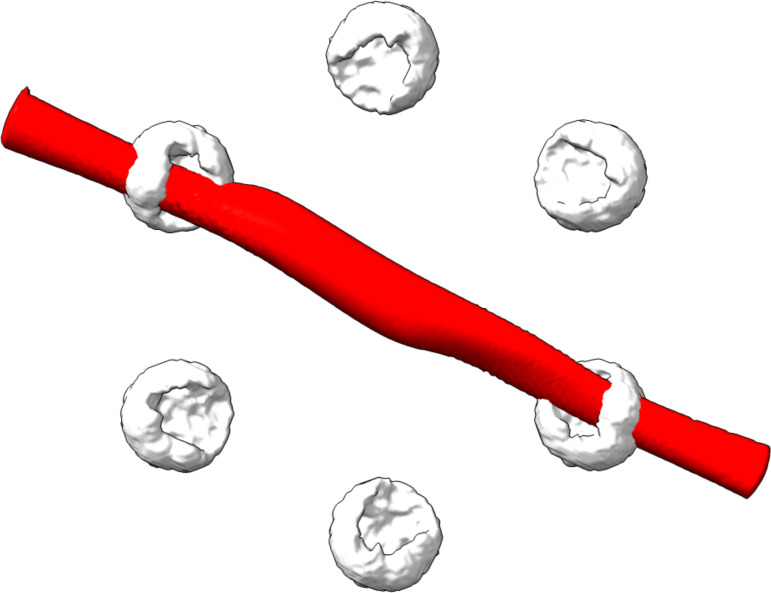
Validation of the particle classification of the hexamer protein ClpB. Here, we took all unclassified 2D images with correlation scores below 0.8 and calculated the 3D reconstruction as described in the method section of main text. The red volume in the image shows no valid structure and could not be fitted to the white clouds which represent the accessible volumes of the fluorophores as calculated for each protomer. Figure 3—figure supplement 4—source data 1.3D reconstituted map of unclassified particles.

**Figure 3—figure supplement 5.**
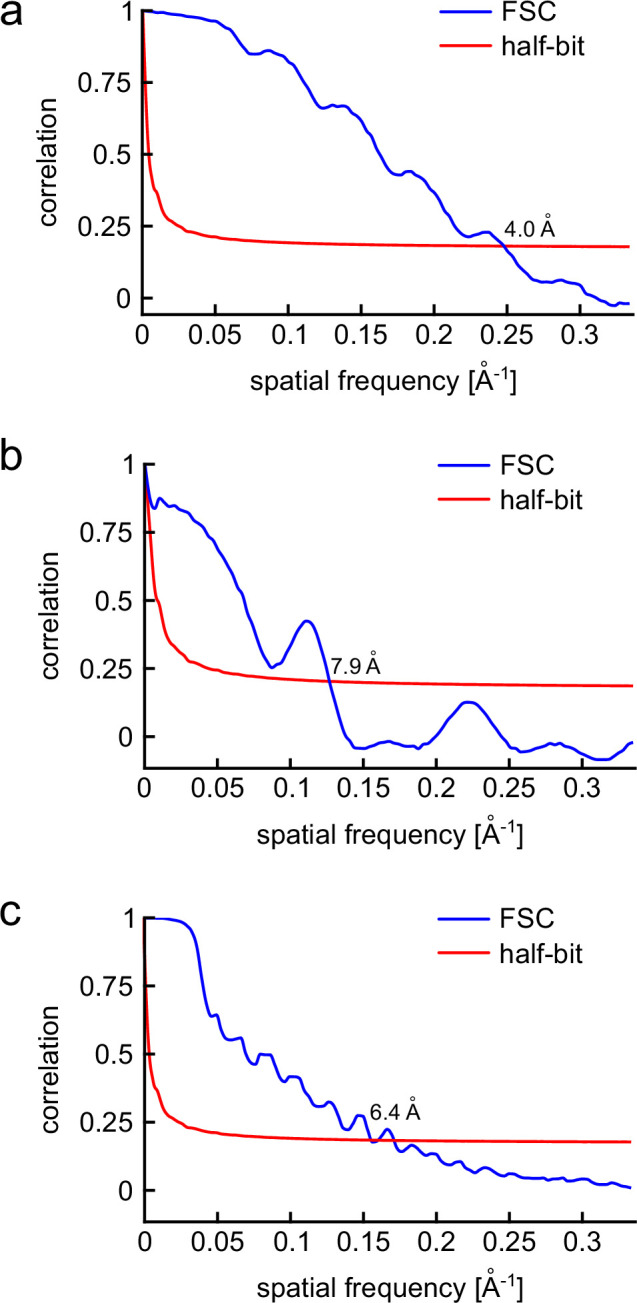
FSC of the 3D volumes obtained for each class. (**a–c**) For each class, an equal number of 2D images were divided randomly into two halves. Then, a 3D reconstruction was calculated for each half-data set, using a low-pass filtered model as an initial guess. The correlation between the two 3D reconstructions is calculated over shells of the Fourier transform and plotted as a function of spatial frequency (blue curves). The spatial resolution of the 3D reconstruction is then evaluated using the half-bit criterion (red solid line) as described in detail in van Heel and Schatz, 2005. (**a**) The FSC of class I yields a resolution of 4.0 Å. (**b**) The FSC of class II yields a resolution of 7.9 Å. (**c**) The FSC of class III yields a resolution of 6.4 Å. Figure 3—figure supplement 5—source data 1.Fourier shell correlation data.

**Animation 2.** *Thermus thermophilus* ClpB 3D reconstruction (side view).

**Animation 3.** *Thermus thermophilus* ClpB 3D reconstruction (top view).

**Figure 4.**
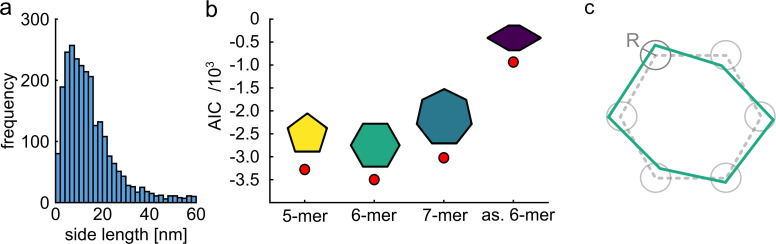
Quantitative model selection for classification. (**a**) Histogram of pair-wise distances from particles with two polarization states, showing a clear peak at ~9 nm as the most probable side length of the ClpB molecules. (**b**) Based on the identified most probable side length we built models of the oligomer with different symmetries, but sharing the same side length of 9 nm, and performed single-particle classification of the experimental images. The AIC criterion shows that the hexamer is the best model. Reducing the symmetry of the hexamer results in a worse fit. (**c**) Schematic of a model deviating from perfect symmetry. Each corner is allowed to be shifted within a circle of radius R. The classification procedure remains accurate for *R* ≤ 1 nm. Figure 4—source data 1.Distance histogram of the hexamer complex for Figure 4a.

An efficient classification benefits from some prior knowledge of the structure. To examine the applicability of our method for samples with unknown symmetry or side lengths, we followed a similar procedure as demonstrated by Curd et al., 2021. First, by inspecting the raw distance histogram of our unclassified particles obtained from single distance measurements (two polarization states), we could identify a peak at ~9 nm as the most probable side length of our molecules (see Figure 4a). Next, we assumed three models with different symmetries for pentamers, hexamers and heptamers, but all sharing the same side length of 9 nm. We simulated an equivalent number of projections for each model and correlated them with our experimental data. Then, we used the Akaike information criterion (Portet, 2020; Curd et al., 2021) to find the best model that describes our experimental data (see Materials and methods section). As shown in Figure 4b, we found that the hexamer structure matches our data significantly better than the other models. In addition, we examined a different case of hexamer model with reduced symmetry and found that this model resulted in a considerably worse fit to our data (Figure 4b).

**Figure 4—figure supplement 1.**
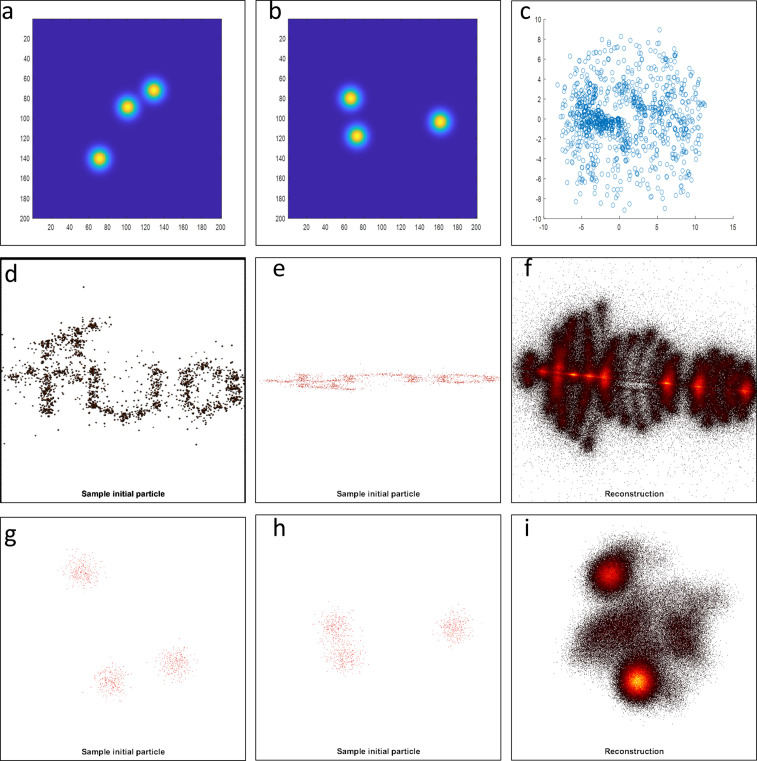
Testing different algorithms for template-free particle reconstruction. Here we tested the code published by Heydarian et al., 2018 which is a template-free particle-fusion based on all-to-all registration approach. (**a–b**) First, we fed the coordinates of our 2D super-resolved experimental images obtained from the set of 467 particles used to generate the hexamer structure into the algorithm. Our experimental data samples different 3D orientations. Examples of different initial 2D projections are shown. (**c**) No clear reconstruction was obtained. (**d-e**) To explore this code further, we simulated 3D rotations of the structure ‘tud_ralf’ used in their study with 100% labeling efficiency. We applied rotations around the x axis within ±180 degrees (i.e. not only in-plane rotations as in the original code) and used the code ‘all-to-all’ to solve the structure. The panels show examples of different initial arrangements for two different orientations. (**f**) No clear reconstruction was obtained. (**g–h**) Last, we also used a simulated data set of our own hexamer structure. Here, we simulated 500 particles with 900 localization each, with a localization precision below 1 nm, a labeling efficiency of 50%, and different 3D orientations in order to mimic our experimental conditions. (**i**) Again, the algorithm fails to deliver a clear reconstruction.

**Figure 4—figure supplement 2.**
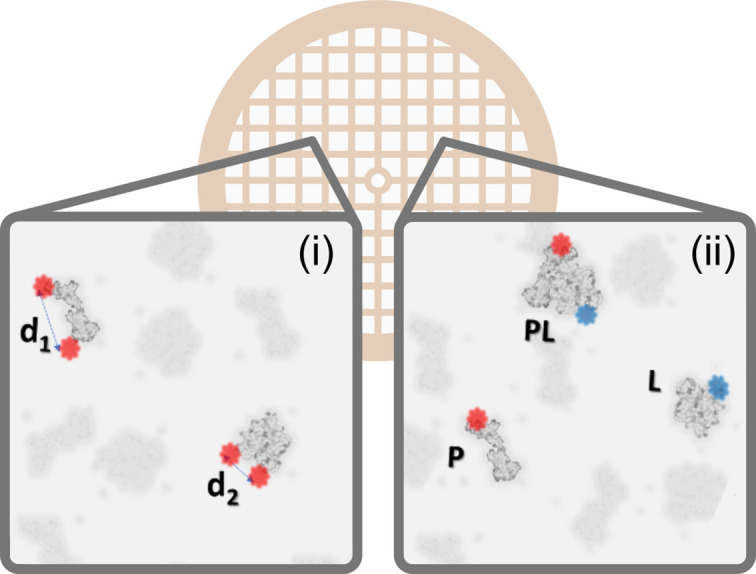
Correlative polarCOLD and cryoEM on the single particle level. By measuring the polarization signal of multiple fluorophores one can classify the particles in a low-contrast EM sample based on distances and orientation. In the exemplary case on the left (i) molecules are identified based on intermolecular or intramolecular distances, indicated by d_1_ and d_2_. The case on the right (ii) illustrates the sorting and sizing of interacting molecules using nanometer distance measurement with multi-color polarCOLD, where Apo proteins (P) and ligand (L) are labeled with different fluorophores, such that the resulting complex (PL) exhibits fluorescence at both wavelengths.

Quantification of the robustness of our approach for samples without symmetry and order goes beyond the scope of our current study, but one simple strategy would be to consider a symmetric structure such as a hexamer and allow for each corner to deviate within a circle of radius R (see Figure 4c). Simulations show that classification of our current data becomes less robust for *R*>1 nm. We point out that solving a completely disordered structure without any prior knowledge would only be possible for complete labeling.

Classification and reconstruction have recently been applied to other single-molecule localization microscopy studies, and in some cases specific algorithms have been developed to handle some degree of partial labeling (Heydarian et al., 2018; Sieben et al., 2018; Salas et al., 2017). As shown in Figure 4—figure supplement 1, however, the existing algorithms do not provide a satisfactory solution for our data. We attribute this to the fact that the structures in our investigations are randomly oriented in 3D, are strongly under-labeled, and involve classes that are similar. A mathematical analysis of the performance of various algorithms for structures of different morphologies is beyond the scope of our current work.

## Discussion

polarCOLD can be further improved and extended to other modalities, for example via combination with genetic labeling (Hoffman et al., 2020; Dahlberg et al., 2018; Liu et al., 2015; Creemers et al., 2000) or by exploiting the narrow absorption and emission spectra at low temperatures for co-localization of larger numbers of fluorescent markers. However, the currently achieved SNR and Angstrom resolution of cryogenic light microscopy is already capable of providing pivotal solutions for quantitative structural analysis of small proteins as well as large protein complexes and aggregates. A specially promising line of study concerns the conformation of membrane proteins in their native environment. Indeed, an estimated 20% of the human genome encodes membrane proteins and many of them are potential drug targets (Piccoli et al., 2013). The structure and active configuration of many proteins and protein complexes, however, remain out of reach because it is very challenging to crystallize or resolve them in their crowded surrounding (Cheng, 2018). In particular, heterogeneities caused by mobile domains and intrinsic distributions in assemblies cannot be preserved and classified in a robust fashion so that they lead to a smearing effect in the final reconstruction in EM studies (Nwanochie and Uversky, 2019; Serna, 2019; Scheres, 2016; Scheres et al., 2007, Orlova and Saibil, 2004; Papai et al., 2020). A future direction is, thus, to vitrify biological samples via rapid freezing (Dubochet and McDowall, 1981) or high-pressure freezing (Studer et al., 2008; Hoffman et al., 2020) in order to exploit the high spatial resolution, sensitivity and specificity of polarCOLD for identifying and determining the positions and orientations of individual particles of interest in low-contrast EM micrographs.

The combination of cryogenic light microscopy with cryoEM has attracted attention for cellular and tissue imaging, although the existing reports do not surpass a resolution of about 10 nm (Dahlberg and Moerner, 2021; Hoffman et al., 2020; Dahlberg et al., 2020; Wu et al., 2020; Tuijtel et al., 2019; Wolff et al., 2016; de Boer et al., 2015; Loussert Fonta and Humbel, 2015; Kaufmann et al., 2014b, Chang et al., 2014; Kaufmann et al., 2014a, Faas et al., 2013; Watanabe et al., 2011; Mironov and Beznoussenko, 2009; Agronskaia et al., 2008; Sartori et al., 2007). A very exciting prospect is now to combine polarCOLD and cryoEM on the single-particle level. As illustrated in Figure 4—figure supplement 2, by labeling a functional domain within a single molecule with two or three fluorophores, it should be possible to classify the particles based on conformational changes as indicated by their intermolecular distances. Similarly, by labeling two different protomers or functional domains within a protein complex, it will be possible to group the particles based on intramolecular distances that result from conformational changes between subunits. In addition, while we currently screen different symmetries to find the best model that fits to the structures under study, we plan to explore unsupervised classification schemes for identifying and classifying particles in an unknown sample. In addition, one can exploit the axial information directly and follow a similar approach by Heydarian et al., 2021. Altogether, these approaches will help to overcome various challenges connected to low purification yields and large backgrounds from cell membranes, paving the way to studying samples containing a heterogeneous distribution of similar structures and mapping their energy landscapes.

## Materials and methods

**Key resources table:** 

Reagent type(species) or resource	Designation	Source or reference	Identifiers	Additional information
Peptide, recombinant protein	PCNA	Sigma Aldrich	Cat. #: SRP5117	
Peptide, recombinant protein	ClpB	https://doi.org/10.1038/s41467-019-09474-6		
Chemical compound, drug	ATTO647N Ni-NTA	Sigma Aldrich	Cat. #: 02175–250 UG-F	
Chemical compound, drug	ATTO647N Maleimide	Sigma Aldrich	Cat. #: 05316–1 MG-F	
Chemical compound, drug	Poly-vinyl alcohol (PVA)	Sigma Aldrich	Cat. #: 360,627	
Chemical compound, drug	6-Hydroxy-2,5,7,8-tetramethylchroman-2-carboxylic Acid	TCI Deutschland GmbH	Cat. #: H0726	
Software, algorithm	MATLAB 2020a	MathWorks		
Software, algorithm	UCSF ChimeraX	http://www.rbvi.ucsf.edu/chimerax		
Software, algorithm	DISC	DOI: 10.7554/eLife.53357		
Software, algorithm	3D reconstruction	https://doi.org/10.1016/j.jsb.2015.03.009		
Software, algorithm	FSC	https://www.ebi.ac.uk/emdb/validation/fsc/		

### Protein labeling

His-tagged human PCNA protein was purchased from Sigma Aldrich (catalogue number SRP5117), at a concentration of 6.6 µM. The protein was specifically labeled via the histidine linker on the N-terminal side of the protein with the dye ATTO647N containing a Ni-NTA functional group which was also purchased from Sigma Aldrich (catalogue number 02175–250 UG-F). First, the protein was desalted to a labeling buffer (25 mM HEPES, 25 mM KCl, pH 7.8) using a 7 K MWCO Zeba desalting column (ThermoFischer, cat. 89882) and then reacted with dyes at a ratio of 1:4 for 2 hr at RT. The protein was then desalted from the excess of dyes using the same desalting column. The labeling efficiency was estimated using an absorption spectrometer (Nanodrop 2000, ThermoFischer) confirming ~100% labeling efficiency. SDS-page and native gel indicated that the protein is indeed assembled and labeled. N-terminal His-tagged ClpB protein, mutated at residue 428 from alanine to cysteine, was purified as described in a previous publication (Mazal et al., 2019). The labeling of the single cysteine was done following the same procedure as describe above with ATTO647N maleimide as a specific labeling agent of the cysteine. Absorption spectroscopy, SDS-page and native gel indicate complete assembly of the protein, see previous publication (Mazal et al., 2019).

### Sample preparation

Both protein samples were prepared in a similar way, and all the samples were prepared freshly on the same day. Proteins were diluted to a stock solution of 50 nM, in 25 mM HEPES, 25 mM KCl, 10 mM MgCl_2,_ 0.5 mM TCEP at pH 7.8 (working buffer). In the case of ClpB 2 mM ATP was added to stabilize protein assembly. The stock solution of poly-vinyl alcohol (PVA) was prepared as follows: 15 µl of 8% PVA (0.3% final concentration) was diluted into 345 µl working buffer containing 1 mM Trolox and 2 mM ATP in the case of ClpB. Then 2 µl of the protein was mixed with the 360 µl PVA solution and filtered with a 100 nm spin filter (Whatman Anotop-10). 5 µl of this mixed solution was then spin coated (30 s at 1000 rpm followed by 3000 rpm for 60 s) onto a plasma-cleaned mirror-enhanced substrate, prepared in house (Böning et al., 2021), and immediately loaded into our custom-built cryogenic microscope.

### Experimental setup

All experiments were performed in a cryogenic microscope that is built around a Janis ST-500 flow cryostat and operates at liquid helium temperature. Samples are loaded onto a cold finger and imaged by a 0.95 NA objective (Olympus MPLAPO 100 x), which is mounted in vacuum, onto two separate EMCCD cameras (Andor iXon) in a polarization-resolved configuration. The field of view spans 211 × 313 pixels with a pixel size of 190 nm. A more detailed description of the optical setup can be found in Böning et al., 2021. The laser intensity used in all experiments was set to ~1 kW/cm^2^ and images were recorded with 14 ms exposure time, more than five times faster than the typical off-time. Therefore, we are able to capture individual bursts and minimize the probability of overlapping emission from two or more fluorophores in a single frame. For each field of view, we collected a total of 50,000 or 100,000 frames for PCNA and ClpB, respectively.

### Image analysis

We analyzed raw image stacks from two polarization channels with custom-written MATLAB software. Briefly, we perform dual-channel localization using maximum-likelihood estimation with a Gaussian PSF model. The minor asymmetry due to the 3D dipole orientation causes a small bias of less than 3 nm on average after filtering the data. Mechanical drift during the long acquisition times is first corrected based on image cross-correlation. Localized coordinates from both channels are then registered via an affine transformation and a non-linear correction. Registered PSFs are grouped and their polarization is calculated from the intensities. Single fluorophores are identified via this feature and used as fiducial markers to perform additional drift correction and establish a more precise image alignment via an interpolated map. The residual drift is well below 1 nm and the median registration error is less than 2 nm. Polarization time-traces of multi-fluorophore particles are then analyzed in more detail (see Figure 1—figure supplement 3) by a routine that is based on the DISC algorithm (White et al., 2020) to find the polarization states of multi-fluorophore conjugated particles. Once all polarization states are identified, the associated coordinates are averaged by taking their median to generate a 2D super-resolved image. We took particles which were fitted best to three polarization states and used them for further analysis (see Figure 1—source data 2 for statistics).

### Single-molecule 2D image fitting

To fit the 2D maps of the human PCNA, we used the theoretical distances obtained from the structural model of human PCNA (PDB: 1AXC) and then we performed a least-squares fit over rotation angles and translation using a simulated annealing algorithm. Two-dimensional cross-correlation of the experimental and simulated maps yield a score better than 0.92. In the case of the hexamer protein, ClpB, we generated a large space of multiple 2D projections, ~25,000 images for each class, using a rotation matrix over x, y, and z axis with an angular resolution of 10°. The exact distances were evaluated from the real crystal structural model taking the dye linker into account (ClpB model, PDB: 1QVR, and the full assembly model taken from Diemand and Lupas, 2006). Then we performed 2D template matching between experimental data and all the images from the simulated data. Here, we used a 2D cross-correlation algorithm to calculate the similarity index between two images. The correlation score ranges from 0 to 1, where 1 indicates a 100% match. For each experimental image, we select the fit with the highest score as the best fit to our data, and to assign it to a class. Images that were fitted to all classes with a difference below 10% for classes 1 and 3, and 3% for class 2 in the score were excluded from further analysis to avoid smearing (see Figure 3—figure supplement 3).

### Tomography and 3D reconstruction

We selected the particles which were fitted properly to the simulated 2D projections, that is particles with a cross-correlation score better than 0.9 and a localization precision better than 3 nm. The 2D maps of the particles were then normalized (maximum and width) to perform the 3D reconstruction. For a full 3D reconstruction, we used the subspaceEM algorithm (Dvornek et al., 2015) with default settings and 100 runs. The algorithm was initialized with an elliptical Gaussian placed in the center of the volume. The 2D maps of the trimer protein, PCNA, were constructed using a grid size of 120 × 120 with a pixel size of 1.5 Å. In the case of the hexamer protein, the grid size was 200 × 200 with a pixel size of 1.5 Å. The 3D volumes were then processed, fitted to the crystal structures/maps of the dye location and edited using ChimeraX (Goddard et al., 2018). The Fourier shell correlation curves (FSC), were computed using the freely available FSC server, provided by the Protein Data Bank in Europe website (https://www.ebi.ac.uk/pdbe/emdb/validation/fsc/). Here we divide the 2D images into two equally large data sets, and calculate their 3D reconstruction as described above. Then we align the two reconstructed volumes and calculate the FSC and determine the resolution based on half-bit criteria (van Heel and Schatz, 2005).

### Quantitative model selection using the Akaike Information Criterion (AIC)

To show that our method can infer the proper symmetry of a protein complex, we followed a similar approach as demonstrated by Curd et al., 2021, but taking the random projections into account. First, we simulated multiple projections of a regular pentamer, hexamer and heptamer, all with the same side length of 9 nm. Given their symmetry, we obtain 2, 3, and 4 classes when considering three randomly placed fluorophores, respectively. We also simulated a deformed hexamer (as. 6-mer) composed of two isosceles triangles of 9 nm length on opposing sides of a square with a side length of 4 nm. Here, we need to consider a total of 5 classes. Then, we performed 2D cross correlation of the experimental data with these templates, and we picked the particles with a localization precision better than 3 nm and correlation score better than 0.5. The normalized sum of square residuals (nSSR) for each model is calculated as

$$nSSR=\sum_{j=1}^{N}\frac{\sum_{i=1}^{n}{\left(1-x_{i}\right)}^{2}}{n}$$

where j is the number of classes, n is the number of images per class, and x_i_ is the score of the classified image. Then, we assessed how well each model fits the data by calculating its AIC value (Portet, 2020; Curd et al., 2021) via $AIC=Mlog\left(nSSR\right)+2K$, where M is the total number of particles in each model and K is the number of parameters (8, 10, 12, 13 for the pentamer, hexamer, heptamer and deformed hexamer, respectively). K was calculated based on the number of sides in each model, number of distances, and number of classes. For example, the pentamer has 5 sides, 1 distance, and 2 classes.

### Distance fitting

For more quantitative analysis of the pair-wise distances we used a model described in Weisenburger et al., 2017; Böning et al., 2021. In short, we convolve the Rician distribution of the distance between two spots with finite localization uncertainty with the projection onto the image plane given by a cosine function. In the case of the PCNA trimer, we only need to consider a single distance due to the symmetry of the structure.

### Determination of expected measurement yield depending on angular resolution

Our co-localization procedure relies on identifying the unique polarization signature belonging to one of a maximum of N fluorophores within a diffraction limited area. To this end, we can choose to consider the complete distribution of polarization values and identify well-separated peaks (histogram method), or consider polarization information in the time domain and identify recurring levels and transitions between them (DISC algorithm White et al., 2020). Both methods are limited in resolution, that is depending on the signal-to-noise ratio and switching rates there is a smallest difference between any two fluorophore dipole orientations that we are able to resolve. We consider a particle with N fluorophores to be completely resolved if all N-1 pairwise angular separations are above this angular resolution. Thus, the yield of completely resolved particles depends on how well two polarization states can be distinguished. We performed a Monte Carlo simulation to estimate this yield in our measurements. To this end, we generate large sets of N random fluorophore angles in the interval [0°,90°] and compute the pairwise differences. For a given angular resolution s between 0.1° and 90° we then calculate the fraction of particles for which all differences are larger than s. This simple estimation shows that for a case of just 2 fluorophores an angular resolution of 20° is already sufficient to achieve a yield of 50%. The same yield for the case of 6 fluorophores, however, requires an angular resolution of 2°.

## Data Availability

Raw data generated or analyses during this study are included in the manuscript file and supporting files. Source data files have been provided for Figure 1b, c, Figure 1 - figure supplement 1 and 3. Figure 2a, b, c, d, Figure 2 - figure supplement 3. Figure 3a, b, c, Figure 3 - figure supplement 2,4, 5. Figure 4a.

## Additional files

Transparent reporting form
