# Supplementary figures and images for: Deciphering a hexameric protein complex with Angstrom optical resolution
Source: eLife. 2022 May 26;11:e76308. doi: 10.7554/eLife.76308 (PMC9142145; doi:10.7554/eLife.76308)

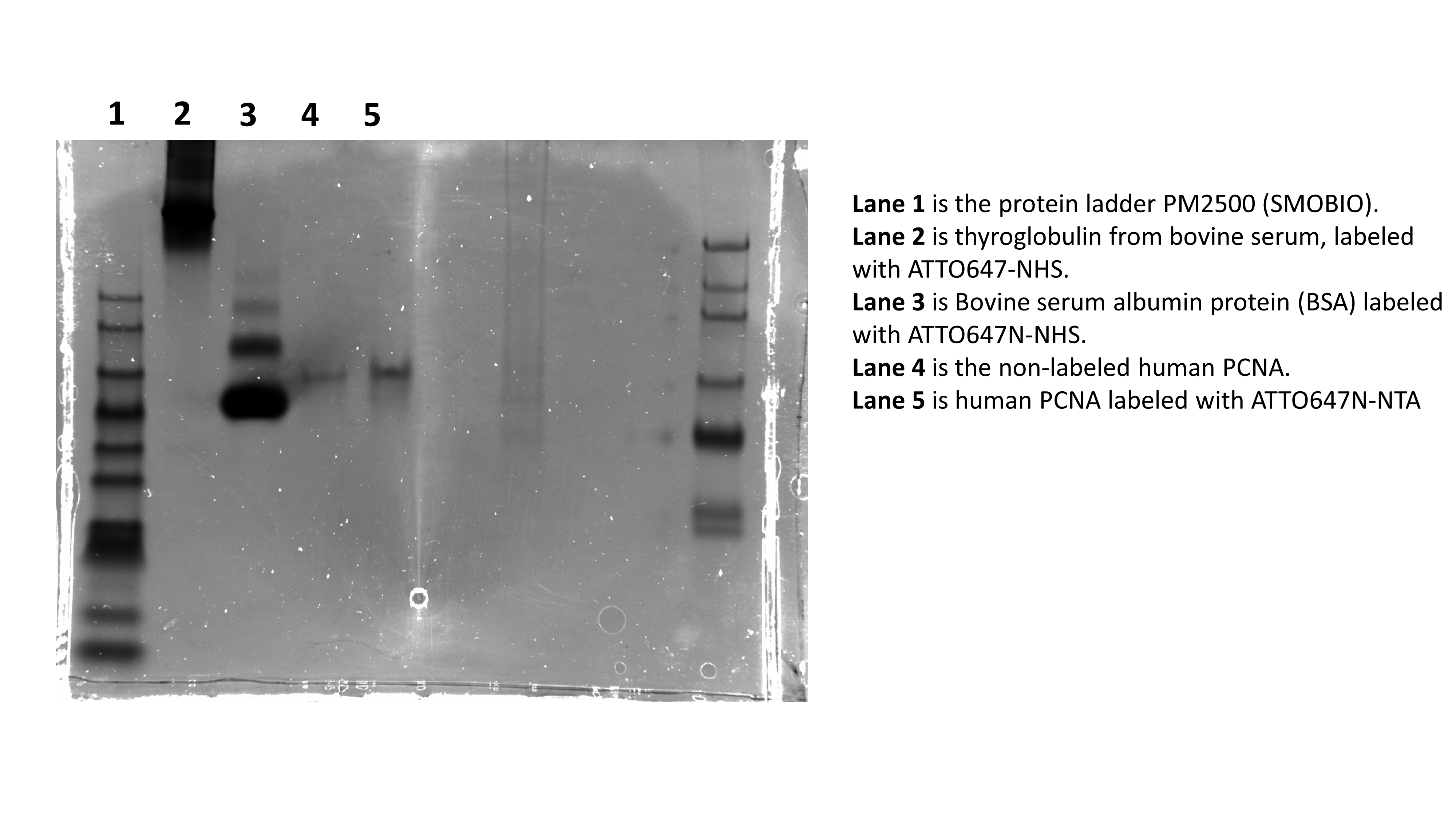

Supplement: Figure 1—figure supplement 1—source data 1. [file elife-76308-fig1-figsupp1-data1.zip › Figure 1-figure supplement 1 -source data 1/Native PAGE_Comassie blue_labeled.tif]

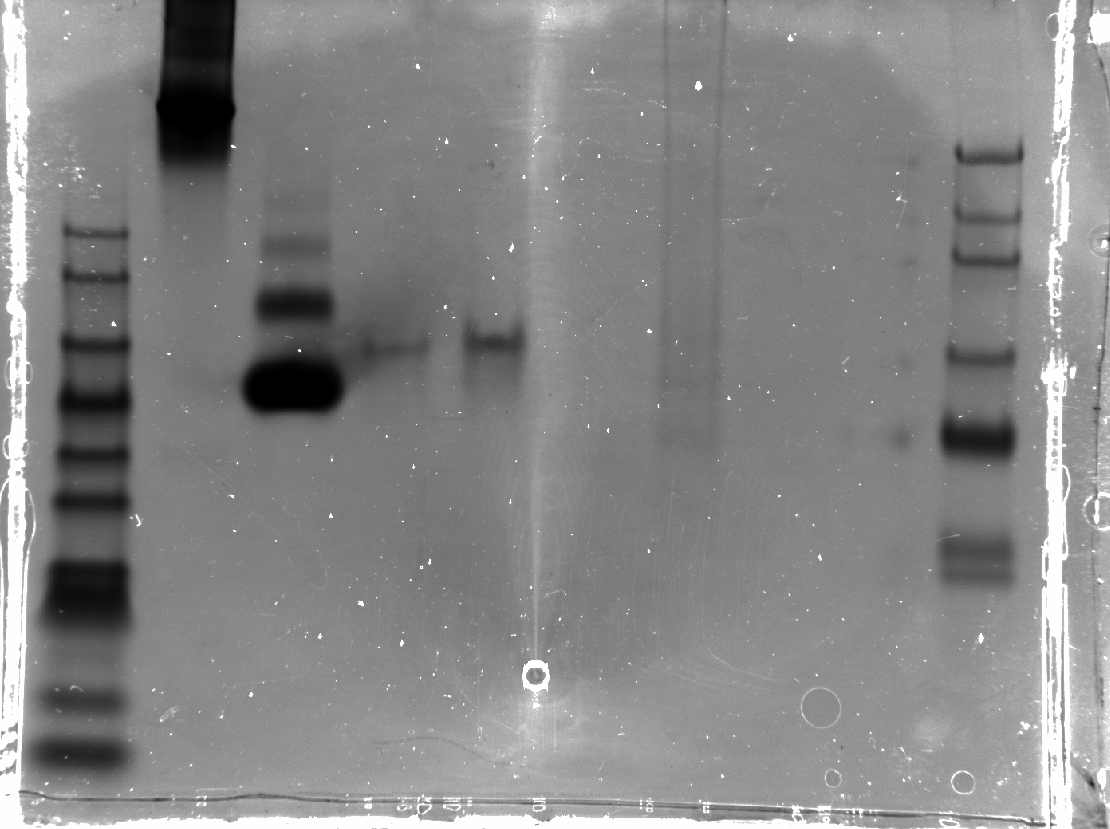

Supplement: Figure 1—figure supplement 1—source data 1. [file elife-76308-fig1-figsupp1-data1.zip › Figure 1-figure supplement 1 -source data 1/Native PAGE_Comassie blue_Raw.tif]

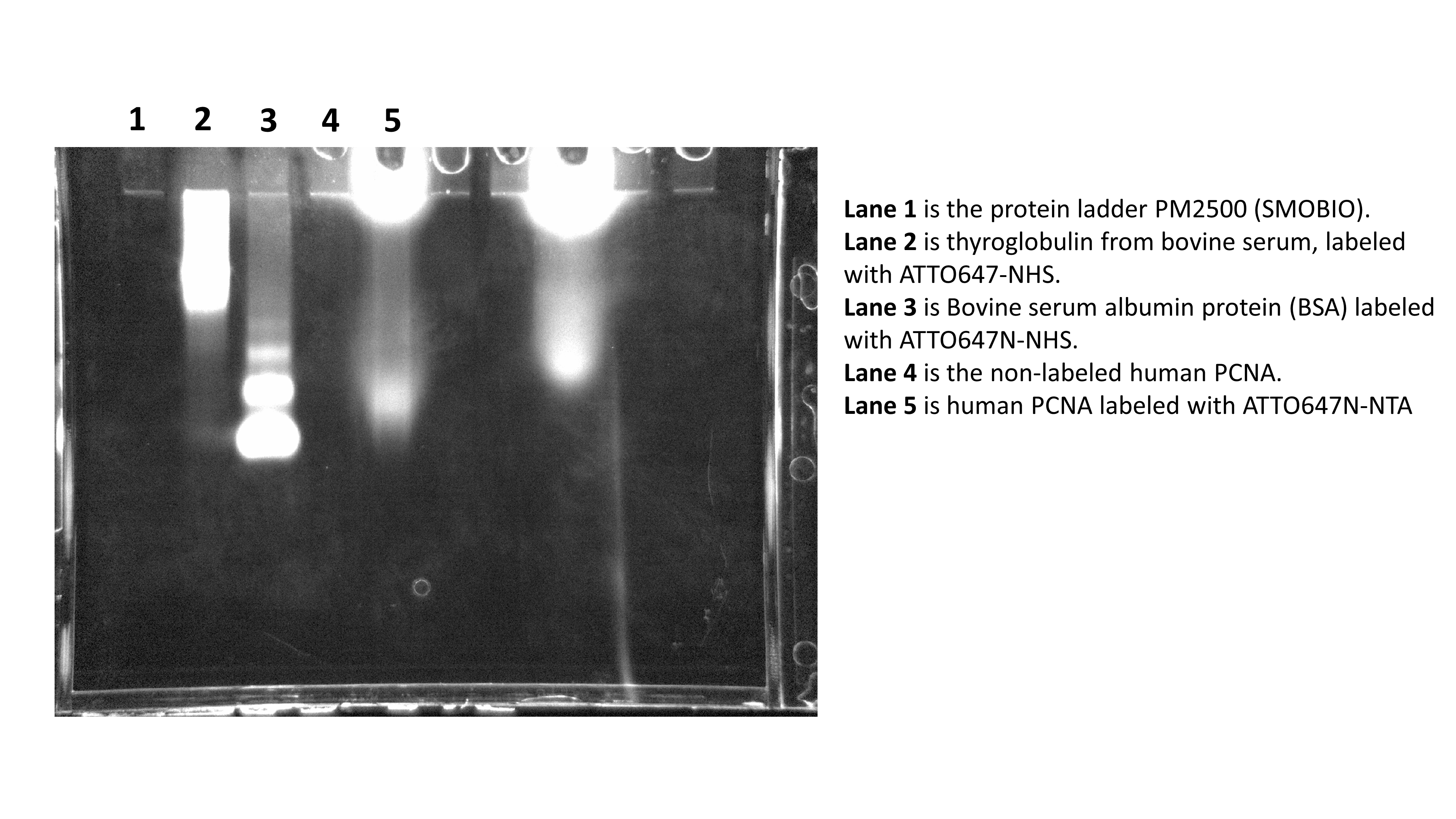

Supplement: Figure 1—figure supplement 1—source data 1. [file elife-76308-fig1-figsupp1-data1.zip › Figure 1-figure supplement 1 -source data 1/Native PAGE_Fluorescence_647 nm channel_Labeled.tif]

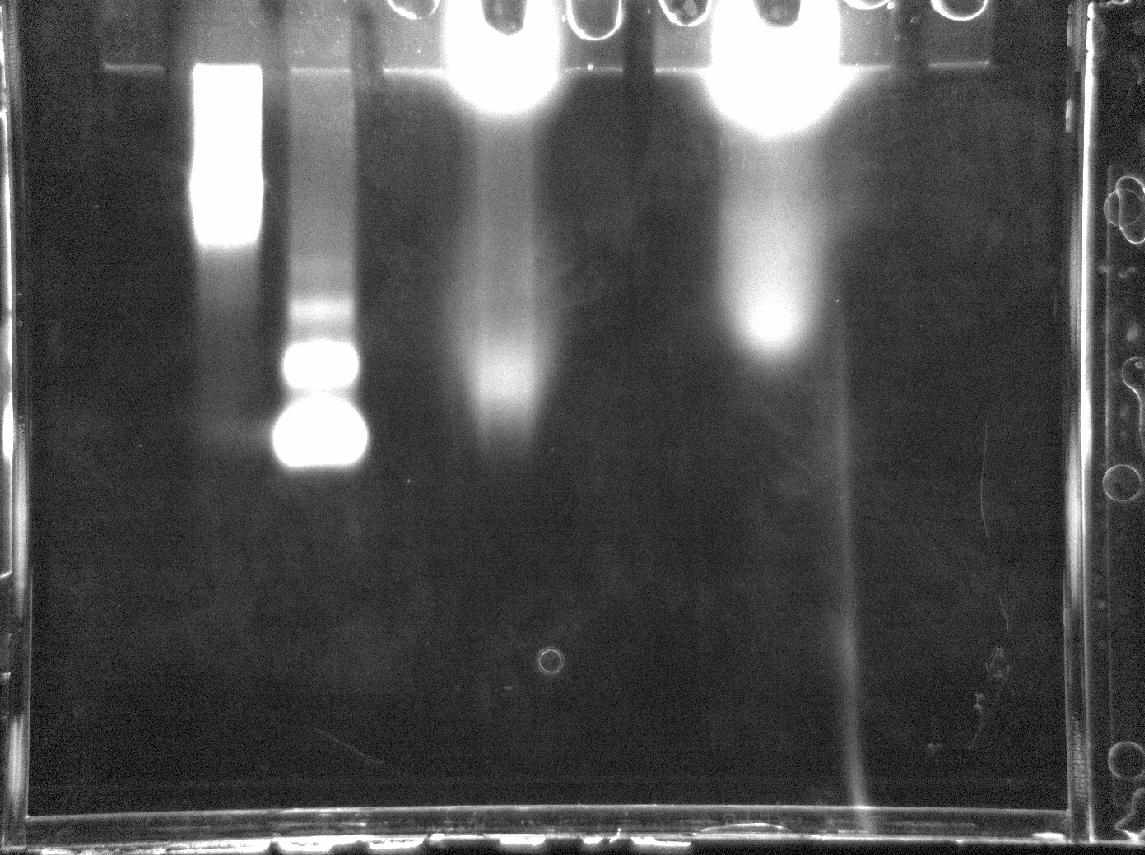

Supplement: Figure 1—figure supplement 1—source data 1. [file elife-76308-fig1-figsupp1-data1.zip › Figure 1-figure supplement 1 -source data 1/Native PAGE_Fluorescence_647 nm channel_Raw.tif]
